# Cataract indicators: their development and use over the last 30 years

**Published:** 2018-02-08

**Authors:** Hans Limburg, Jacqui Ramke

**Affiliations:** 1 Consultant: Public Eye Health Health Information Services, Grootebroek, Netherlands.; 2 Senior Research Fellow: University of Auckland, Auckland, New Zealand.

**How do we know if our cataract service is reaching enough (and the right) people? How can we tell whether the quality of surgery is good enough? Understanding cataract indicators, and how to use them, can help us to meet the community's needs.**

**Figure:**
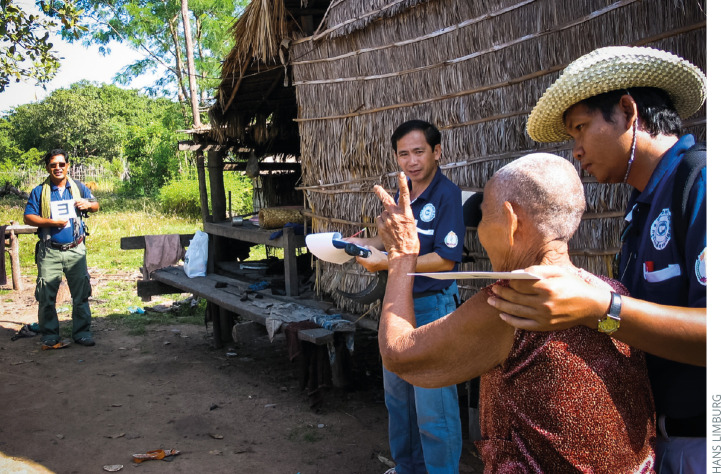
Visual acuity is tested during a RAAB survey. CAMBODIA

For the last four to five decades, cataract has been the most common cause of blindness worldwide; it is also a leading cause of visual impairment. Before this, fewer people grew old enough to develop cataract and infectious diseases (trachoma, onchocerciasis, measles) were thought to cause most blindness. Thanks to improved hygiene, antibiotics, ivermectin, vaccinations and vitamin A distribution, eye infections and xerophthalmia have become less common. At the same time, life expectancy increased, leading to a rapid increase in the incidence (i.e. new cases) of cataract. Because the incidence of cataract increases exponentially with increasing age, the age composition of the population determines the number of new cases of cataract; i.e., countries with older populations tend to have more people with cataract.

Several important advances in cataract surgery took place over the past 30 years, including the advent of microsurgery, the introduction of IOLs, and the transition from intra-capsular to extra-capsular and then small incision surgery. These advances allowed surgery to be undertaken earlier and vastly improved postoperative visual outcomes, which led to an increased demand for services. In parallel with these improvements, the indicators for monitoring services – and the tools to collect monitoring data – have also developed. In this article we describe these developments, and also discuss how to interpret and use indicators to improve cataract services.

## Quantity of cataract surgery

When addressing cataract blindness became a priority, the focus was on increasing the number of cataract operations. **Cataract surgical rate (CSR)** was the first cataract indicator commonly reported. By recording all cataract operations, the total output of cataract operations in a year in a defined population (hospital catchment, district, province or country) can be determined, and trends over time followed.^1^ CSR is obtained when the cataract output in a given year is divided by the number of people (in millions) in the defined area.

**Cataract surgical rate (CSR) = number of cataract operations per million population per year**

It is important to monitor CSR for a whole year rather than part of a year, so any seasonal variations in demand for services are accounted for.

The CSR can be as high as 10,000 in some developed market economies, and less than 1,000 in some countries with a young population and/or inadequate eye care services.

A **target CSR** can be established based on the desired output of the available cataract surgeons in the area, or on the estimated incidence^2^ of operable cataract.

Because the incidence of cataract is lower in countries with a younger population and a lower life expectancy, the target CSR will be lower there as well.

The **number of operations** can be used to set and monitor output targets and compare the efficiency of cataract services and surgeons in different hospitals or geographic areas. For example:

- **Average weekly output per cataract surgeon:** the total number of cataract operations divided by the total number of cataract surgeons in the same area, divided by the number of weeks in a working year.
- **Annual output per cataract surgeon:** the total number of cataract operations per individual cataract surgeon within a 12-month period.

## Quality of cataract surgery

As surgical techniques have advanced, publications from leading eye hospitals reported ever-improving outcomes. However, many eye surgeons worldwide currently work under less favourable conditions and their results are seldom published. In the late 1990s, some population-based surveys showed that up to 40% of operated patients could not see 6/60 after cataract surgery.^3^ In response, the World Health Organization (WHO) published recommendations on the outcome of cataract surgery in 1998 (Table 1). According to the WHO, fewer than 5% of cataract patients should be unable to see 6/60 (best corrected or presenting).^4^

**Table 1:** WHO guidelines on visual outcome of cataract surgery 6–12 weeks post-operatively^4^

Postoperative visual acuity (VA) threshold		Target for the proportion of operated eyes achieving VA thresholds	
		Presenting visual acuity (PVA) or VA with available correction	Best corrected visual acuity (BCVA) or VA with pinhole
**Good**	6/18 or better	>80%	>90%
**Borderline**	<6/18–6/60	<15%	<5%
**Poor**	Worse than 6/60	<5%	<5%

The indicator for the quality of cataract surgery is **cataract surgical outcome (CSO),** which is the visual outcome in the operated eye.

**Cataract surgical outcome (CSO) = visual acuity in the operated eye**

Paper-based and computerised software tools were subsequently developed to monitor cataract surgical outcome on a routine basis.^5,6^ The following information is recorded for each operation:

- Visual acuity (VA) before surgery
- Surgical technique used
- Whether the outcome is good, borderline or poor, both after surgery and at follow-up
- The type of complication, if any
- The major cause of each poor outcome.

The proportion of good, borderline or poor outcomes and the proportion of complications can be calculated. In the software tools, filters can be applied to the dates, surgeon, clinic and other parameters to make more detailed analysis possible. The software is intended to provide insight as to where and how modifications in the service can be made to improve visual outcome further.

The system is definitely not intended to compare individual eye surgeons or clinics, but to monitor improvement in outcome over time for the same surgeon or clinic. Unfortunately, many ophthalmologists have been reluctant to use the monitoring tools available, and we must identify and overcome the barriers to incorporating monitoring of outcomes into routine practice.

## Population-based indicators

As cataract services continued to develop, it became clear that population-based information was needed that could capture local variations in disease pattern, environment and available resources. It was equally important to gather information on the population, rather than only those who were accessing services. Cross-sectional surveys can provide this information on the eye care situation within a defined area, such as a district, province or country. The information can then be used to plan and monitor services.

The rapid assessment of avoidable blindness (RAAB) methodology was specifically developed to collect data that would make it possible to plan eye care services for a population of between 0.5 and 5 million people. RAAB surveys are restricted to those aged 50 years and above, where the prevalence of blindness and visual impairment is highest. As a result, the sample size can be smaller and the survey is faster and less expensive to carry out than traditional full-population surveys.^7^ RAAB software includes standardised and automatic data analysis and reporting. It generates four important cataract indicators (see panel below).

**Cataract indicators generated by RAAB software**

**Prevalence of blindness and visual impairment due to cataract** and **estimated number of cases.** The sample prevalence and age-and sex-adjusted estimates of the cataract burden in the survey area are given.

**Cataract surgical coverage (CSC).**^8^ This is the proportion of people with bilateral cataract who have been operated upon in one or both eyes. Results are given separately to show coverage among people with best corrected visual acuity (BCVA) of <3/60, <6/60 and <6/18. It is written as either CSC_<3/60_, CSC_<6/60_ or CSC_<6/18_ and expressed as a percentage. E.g., ‘CSC_3/60_ 85%’ means that 85% of people with BCVA of 3/60 have had surgery in one or both eyes.

**Cataract surgical outcome (CSO).** The proportion of operated eyes with a good visual outcome (6/18 or better) after cataract surgery, written as CSO_good_, The causes of poor outcome are also given.

**Effective cataract surgical coverage (eCSC)**^9^ is the proportion of people with bilateral cataract and BCVA of <3/60, <6/60 or <6/18 who have received cataract surgery in one or both eyes *and* have postoperative presenting VA of 6/18 or better in at least one operated eye.

Other indicators reported by RAAB include **barriers to surgery** and **details of surgery** (location, type, cost). RAAB also generates information on the **main causes of blindness and VI** which identifies where cataract is positioned in terms of priority for intervention.

## Equity of cataract services: disaggregating indicators

Cataract services are not used equally by people within countries. For example, in many settings high quality cataract surgery is provided to wealthy urban people, often before visual impairment occurs. In contrast, similar services are scarce or absent for the rural poor. If only wealthy urban people receive surgery, a high cataract surgical rate would not automatically mean that the coverage will be high or that the prevalence of cataract blindness will be low.

All cataract indicators can be disaggregated (reported separately) by gender, location (urban or rural), socio-economic status, or disability, for example.^10^ When this is done, inequity is often identified. For example, women and rural dwellers tend to have lower cataract surgical coverage, a higher burden of cataract blindness, and worse postoperative visual outcomes than men and urban dwellers.^11^

Disaggregated cataract indicators are essential in order to understand the nature and extent of inequality in the population, to inform appropriate strategies to reduce inequality, and to monitor whether improvements in services (e.g. quality and access) are experienced by the groups who need them most. Cataract surgical outcome monitoring and RAAB software already present results separately for women and men. In future, disaggregation for other factors (e.g. socio-economic status) should become possible.

## Interpreting cataract indicators

The current global action plan has chosen cataract surgical rate (CSR) and cataract surgical coverage (CSC) as its service delivery indicators, but a clearer picture of cataract services emerges when data are available for a broad range of indicators from both facility-based and population-based sources. Also, rather than considering just CSR and CSC in isolation, they should be considered in combination with other cataract and eye health indicators. For example, a high CSR alone may not reflect ‘good’ cataract services, without also considering the cataract surgical outcomes (CSO) of the operations, who was operated on (to ensure equity), and whether coverage (CSC and effective cataract surgical coverage, or eCSC) is improving. We have provided three scenarios in Table 2 below to demonstrate how helpful it can be to use a range of indicators to identify the specific aspect(s) of the service that require improvement.

## Conclusion

Cataract indicators and monitoring processes have evolved alongside cataract services over the past 30 years and will continue to do so in future. To be useful, indicators require good quality data and careful interpretation by clinicians and programme managers in order to identify which aspects of cataract services are most in need of being strengthened.

**Table 2:** Three scenarios to illustrate the interpretation of various RAAB cataract indicators (for people aged 50 and over)

RAAB indicators	Interpretation
***Scenario 1***	
All blindness: 2.7%; Cataract blindness: 1.2% CSR 1,200 CSO_Good_ PVA 58% (58% of patients had ‘good’ presenting visual acuity, i.e. 6/18 or better. See Table 1.) CSC_<3/60_ 54%; CSC_<3/60_ men 68%; CSC_<3/60_ women 45% eCSC_<3/60_ 39%; eCSC_<3/60_ men 53%; eCSC_<3/60_ women 27% Barriers ‘Not aware’ 32%; ‘Cannot afford’ 25%; ‘Fear’ 25%	The prevalence of blindness due to cataract is moderate/high. CSR is low/moderate. The outcomes of surgery can be improved. CSC and eCSC are moderate/low, and men have considerably better results compared to women.
**Possible response:** This service needs to understand why those who are blind and vision impaired are not undergoing surgery i.e. who they are, where they are and how to deliver services in a more appropriate and accessible way. Health education, improvement of cataract surgical outcomes and more affordable services may increase CSR and CSC.	
***Scenario 2***	
All blindness: 1.8%; Cataract blindness: 0.9% CSR 4,000 CSO_Good_ PVA: 65%; CSO_Good_ PVA women: 47%; CSO_Good_ PVA men: 77% CSC_<3/60_ 82%; CSC_<6/60_ 51%; CSC_<6/18_ 29%	Cataract blindness is moderate. CSR is moderate/good. The proportion of operations resulting in a good visual outcome (CSO_Good)_ is only 65%, compared to a target of 80%. Among women, it is only 47%, compared to 77% among men. Cataract surgical coverage is acceptable at BCVA <3/60. Not much surgery is done at BCVA <6/60 and BCVA <6/18.
**Possible response:** Implement strategies to improve the quality of post-operative vision, particularly among women.	
***Scenario 3***	
All blindness: 0.9%; Cataract blindness: 0.2% CSR 8,000 CSO_Good_ PVA80% CSC_<3/60_ 96%; CSC_<6/60_ 94%; CSC_<6/18_ 81% CSC_<3/60_ richest quintile 99%; poorest quintile 84% CSC_<3/60_ urban 98%; rural 86% CSC_<3/60_ men 99%; women 85%	CSR is high, prevalence of blindness is low and cataract blindness <25% of all blindness. Surgical outcomes are good and overall coverage is high. Cataract seems well under control. However, when CSC is disaggregated by socio-economic status, domicile women experiencing lower coverage compared to richer people, urban dwellers and men.
**Possible response:** Maintain output and quality while implementing strategies to make services more accessible for poorer people, rural dwellers and women.	
